# The effects of cognitive impairment on the multi-scale dynamics of standing postural control during visual-search in older men

**DOI:** 10.3389/fnagi.2023.1068316

**Published:** 2023-01-25

**Authors:** Junhong Zhou, Brad Manor, John Riley McCarten, Michael G. Wade, Azizah J. Jor’dan

**Affiliations:** ^1^Hinda and Arthur Marcus Institute for Aging Research, Hebrew SeniorLife, Boston, MA, United States; ^2^Harvard Medical School, Boston, MA, United States; ^3^Geriatric Research Education and Clinical Center (GRECC), Veterans Affairs Health Care System, Minneapolis, MN, United States; ^4^School of Medicine, University of Minnesota, Minneapolis, MN, United States; ^5^School of Kinesiology, University of Minnesota, Minneapolis, MN, United States; ^6^Department of Exercise and Health Sciences, Manning College of Nursing and Health Sciences, University of Massachusetts Boston, Boston, MA, United States

**Keywords:** cognitive impairment, visual-search, multiscale entropy, dual task, standing postural control, sway complexity

## Abstract

**Background:**

Cognitive impairment disrupts postural control, particularly when standing while performing an unrelated cognitive task (i.e., dual-tasking). The temporal dynamics of standing postural sway are “complex,” and such complexity may reflect the capacity of the postural control system to adapt to task demands. We aimed to characterize the impact of cognitive impairment on such sway complexity in older adults.

**Methods:**

Forty-nine older adult males (Alzheimer’s disease (AD): *n* = 21; mild cognitive impairment (MCI): *n* = 13; cognitively-intact: *n* = 15) completed two 60-s standing trials in each of single-task and visual-search dual-task conditions. In the dual-task condition, participants were instructed to count the frequency of a designated letter in a block of letters projected on screen. The sway complexity of center-of-pressure fluctuations in anterior–posterior (AP) and medial-lateral (ML) direction was quantified using multiscale entropy. The dual-task cost to complexity was obtained by calculating the percent change of complexity from single- to dual-task condition.

**Results:**

Repeated-measures ANOVAs revealed significant main effects of group (*F* > 4.8, *p* < 0.01) and condition (*F* = 7.7, *p* < 0.007) on both AP and ML sway complexity; and significant interaction between group and condition for ML sway complexity (*F* = 3.7, *p* = 0.03). The AD group had the lowest dual-task ML complexity, as well as greater dual-task cost to ML (*p* = 0.03) compared to the other two groups. Visual-search task accuracy was correlated with ML sway complexity in the dual-task condition (*r* = 0.42, *p* = 0.007), and the dual-task cost to ML sway complexity (*r* = 0.39, *p* = 0.01) across all participants.

**Conclusion:**

AD-related cognitive impairment was associated with a greater relative reduction in postural sway complexity from single- to dual-tasking. Sway complexity appears to be sensitive to the impact of cognitive impairment on standing postural control.

## Introduction

Maintaining balance when standing is fundamental for many activities of daily living. Standing postural control in everyday life is often performed with a concurrent cognitive task (e.g., reading or talking). Such “dual-tasking” often negatively impacts performance on the concurrent cognitive task, response of the postural system, or both, which is in turn interpreted as the dual-task “cost.” This dual-task cost is worse in populations suffering from age-related conditions, specifically those with cognitive impairment [e.g., mild cognitive impairment (MCI), dementia or Alzheimer’s disease (AD; Hauer et al., 2003; Manckoundia et al., 2006; Jor’dan et al., 2015; Ghai et al., 2017; Mesbah et al., 2017)]. Laboratory studies have shown that compared to cognitively-intact older adults, those with cognitive impairments oftentimes exhibit increased standing postural sway (e.g., greater sway area), as well as greater dual-task cost to postural sway induced by simultaneous performance of verbalized serial subtractions (Hauer et al., 2003; Manckoundia et al., 2006; Mesbah et al., 2017). These alterations have been associated with greater fall risk in this population (Hauer et al., 2003; Muir-Hunter et al., 2014). Such observations necessitate further characterization of standing postural control in older adults with cognitive impairment, especially during more ecologically valid dual-task conditions. Knowledge gained may provide critical insight into the underlying mechanisms through which cognitive impairment disrupts postural control and thus increases fall risk in this vulnerable older adult population.

Traditionally, standing postural control has been characterized by temporospatial sway metrics including center-of-pressure (COP) sway area, or path length. These metrics are derived from a single scale of time or space. However, standing postural control is regulated by multiple neurophysiologic processes that include the functional integration of multiple sensory inputs (e.g., visual and vestibular inputs), spinal and supraspinal neural networks, the peripheral neuromuscular system, and a host of cognitive functions (e.g., attention). These elements are dynamic and interact with one another over multiple, not single, scales of time and space (Peterka, 2002). As such, the dynamics of postural sway COP fluctuations are complex; that is, they contain meaningful information over multiple scales that can be captured by metrics derived from complexity theory such as multi-scale entropy (MSE), but not by traditional single-scale metrics. The effects of cognitive impairment on the complexity of standing postural control dynamics under single- and dual-task conditions are relatively unclear. Therefore, we leveraged the data from a previously completed study to address this gap in the literature (Jor’dan et al., 2015).

The primary aim of this analysis was to characterize the impact of conditions related to cognitive impairment (i.e., MCI and AD) on postural sway complexity during single- and dual-task standing. An attention-based visual-search dual-task was implemented to assess the allocation of attention and coordination of perceptual-motor information while also increasing the ecological dual-task validity (i.e., to mimic standing and reading within the environment; Stoffregen et al., 2000). Our primary hypothesis is that compared to cognitively-intact older adults, those with MCI or AD would exhibit lower sway complexity, and such decrease in sway complexity would be even greater in the dual-task condition. Secondarily, we hypothesize that those with poorer capacity to adapt to the “stressor” (i.e., additional cognitive task), as captured by lower sway complexity in the dual-task condition, would have poorer task performance (e.g., lower visual-search task accuracy).

## Methods

### Participants

In the parent study, 53 community-dwelling older adult men with varying levels of cognitive status (i.e., cognitively-intact, MCI, and AD) were recruited from the Veteran Affairs (VA) Medical Center in Minneapolis, MN. Potential participants were screened by a clinical team, consisting of a neurologist, geriatric psychiatrist, internist, and a neuropsychologist, for cognitive decline within a 4-month period prior to study initiation (Jor’dan et al., 2015). For the current analysis, the inclusion criteria included being aged 60–90 years and able to stand without assistance for at least 60 s. The exclusion criteria included any other neurological or musculoskeletal disorders, physician-diagnosis of peripheral neuropathy that is associated with diminished standing postural control, and impaired visual acuity as assessed by the inability to see presented letters within the visual-search task. All participants or their guardians provided informed consent approved by the Institutional Review Board of the Minneapolis VA Medical Center.

### Postural control assessment

#### Apparatus

Standing postural sway was recorded using a standard Wii balance board (Nintendo, Inc.) (Koslucher et al., 2012; Scaglioni-Solano and Aragón-Vargas, 2014). The time series of COP fluctuations were recorded in the anterior–posterior (AP) and medial-lateral (ML) direction at 30 Hz.

#### Protocol

The measurement of postural sway variability during performance of a visual-search task in older adults with and without AD has been previously published in detail (Jor’dan et al., 2015). Briefly, target boards (33.75 cm × 42.5 cm) were placed 2.5 m in front of the Wii board at the eye level of each participant. Participants completed two 60-s trials of quiet standing (i.e., single-task, control) and dual-task standing (i.e., visual-search) conditions in block design (Stoffregen et al., 2000; Prado et al., 2007; Chang et al., 2010; Koslucher et al., 2012; Jor’dan et al., 2015). For the single-task standing condition, participants were instructed to stand comfortably while keeping their gaze within the blank white target board. For the visual-search dual-task, participants were presented with a target board comprised of 156 randomized alphabet letters in a grid. Participants were instructed to count the frequency of a designated letter (i.e., M and T, one for each trial). If the participant finished counting before the end of the 60-s trial, participants were instructed to go back and “check their work.” At the end of each trial, participants reported their count of the designated letter.

### Data analysis

#### Postural sway metrics

The COP times-series of the first successfully completed trial (as defined by completing a standing trial without significant disturbance such as talking, turning the upper body, adjusting arms, etc.) per condition was used for the analysis to minimize physical and mental fatigue within the data. The COP postural sway was used to compute traditional sway metrics (i.e., sway path length and sway area).

The sampling frequency of the COP time-series was 30 Hz. To quantify postural sway complexity, MSE analysis was completed on both the AP and ML COP time-series. Empirical mode decomposition (EMD) was used to first remove high-frequency noise and low-frequency trends in the raw time-series (Huang et al., 1998; Zhou et al., 2016). Specifically, we followed the well-established parameters to remove fluctuations at frequencies over 20 Hz as they are noisy and unlikely to reflect physiologically meaningful control processes (Zhou et al., 2016). Fluctuations at frequencies less than 0.2 Hz were also removed to ensure that a sufficient number of dynamic patterns were included in the entropy estimation. EMD-filtered time series were then “coarse-grained” to capture the dynamics on different scales of time. This procedure split the COP time-series into non-overlapping windows of length equaling a scale factor, τ, ranging from 1 to 8 data points. We then computed the sample entropy of each coarse-grained time-series by choosing *m* = 2 and *r* = 15% based on previous recommendations (Gow et al., 2015). The postural sway complexity metric was then computed as the area under the MSE curve, such that smaller areas reflect lower sample entropy values over multiple time scales and thus, lower complexity.

#### Dual-task cost to postural sway metrics

The dual-task cost to the postural sway metrics (i.e., AP and ML complexity, sway path length, and sway area) was quantified as the percent change in each metric from the control to the visual-search condition using the following formula:


$$\frac{visual-searchswaymetric-controlswaymetric}{controlswaymetric}\times 100$$

#### Visual-search task performance

The task performance was calculated as the percent accuracy, that is, the number of target letters reported by the participant divided by the total number of target letters within the amount of letters scanned, multiplied by 100.

### Statistical analyses

Descriptive statistics were used to summarize participant characteristics. Outcomes have been expressed as mean ± SD. Student’s *t*-tests were used to compare these characteristics between groups (i.e., cognitively-intact, MCI, AD).

#### Primary analyses

To examine the effect of cognitive impairment and task condition on the complexity of postural sway, we used repeated-measures analysis of variance (rANOVA). The dependent variable was sway complexity in the AP or ML direction. Model effects were group (cognitively-intact, MCI, AD), condition (control, visual-search), and their interaction. Tukey’s *post-hoc* testing was used for significant models.

To determine the effect of cognitive impairment on the dual-task cost to sway complexity (i.e., the percent change in sway complexity from single- to dual-task standing), we used one-way ANOVA models. The dependent variables were the dual-task cost to AP or ML complexity, and the model effect was group (i.e., cognitively-intact, MCI, AD).

To examine the hypothesis that postural sway complexity in the dual-task condition and their respective dual-task costs would be associated with visual-search task performance (as assessed by the percent accuracy), we used linear regression models.

#### Secondary analyses

To determine (1) the effects of cognitive impairment and task condition on traditional sway metrics, and (2) the effect of cognitive impairment on the dual-task cost to traditional sway metrics, we used similar rANOVA and one-way ANOVA models, respectively, as described above. The dependent variables were sway path length or sway area, and their respective dual-task cost. Additionally, we used similar linear regression to examine the association between these traditional sway metrics during the dual-task condition, their respective dual-task cost and visual-search task performance.

Significance levels were set to *p* < 0.05. Age, education, status of type 2 diabetes and hypertension were included as the covariates for all models described above. Analyses were performed using JMP 16 Software (SAS Institute, Cary, NC, United States).

## Results

### Participant characteristics

Four participants (cognitively-intact =2, AD =2) were excluded from the analysis due to poor quality of postural sway recordings (e.g., poor signal-to-noise ratio or large extraneous body movements during the majority of trials). The remaining 49 participants (cognitively-intact: *n* = 15; MCI: *n* = 13; AD: *n* = 21) were all male, with a mean age of 78 ± 6 years. Groups did not differ in age, BMI, education, or the presence of type 2 diabetes or hypertension (Table 1), and all the participants reported being able to see the presented visual-search letters. All the outcomes of postural sway were normally distributed (Table 2).

**Table 1:** Group demographic and clinical characteristics.

Characteristics	Cognitively-intact	MCI	AD	*p* value
*N*	15	13	21	
Age (years)	78 ± 4	76 ± 7	77 ± 8	0.66
BMI, kg/m^2^	27 ± 3	27 ± 4	26 ± 4	0.37
Education (years)	13 ± 2	14 ± 3	13 ± 2	0.11
MMSE	29 ± 1	27 ± 2	21 ± 5	<0.001*
Type 2 diabetes (% yes)	31	15	21	0.63
Hypertension (% yes)	62	54	63	0.86
Accuracy (%)	92 ± 11	90 ± 17	84 ± 11	0.24

MMSE, Mini-mental State Examination; Data = means ± SD; *p value < 0.05.

**Table 2:** The postural sway metrics in each group.

Postural sway metrics	Cognitively-intact	MCI	AD	*p* value
Postural sway complexity				
Control				
Medial-lateral	9.6 ± 2.0	8.0 ± 2.4	8.6 ± 2.2	0.04*
Anterior–posterior	10.3 ± 2.0	7.5 ± 1.8	8.4 ± 2.3	0.28
Visual-search				
Medial-lateral	8.5 ± 2.0	7.2 ± 1.7	7.9 ± 2.5	0.01*
Anterior–posterior	7.9 ± 1.7	7.2 ± 1.7	6.4 ± 2.5	0.25
Traditional sway measures				
Control				
Sway path length	0.96 ± 0.26	1.15 ± 0.3	1.32 ± 0.5	0.11
Sway area	0.1 ± 0.6	0.21 ± 0.13	0.25 ± 0.3	0.24
Visual-search				
Sway path length	0.9 ± 0.25	0.92 ± 0.24	1.38 ± 0.4	0.002*
Sway area	0.11 ± 0.08	0.11 ± 0.06	0.35 ± 0.34	0.02*

Data = means ± SD; *p value < 0.05; *p* values were from the repeated measures ANOVA models.

### The effect of cognitive impairment and task condition on postural sway complexity

**Figure 1:**
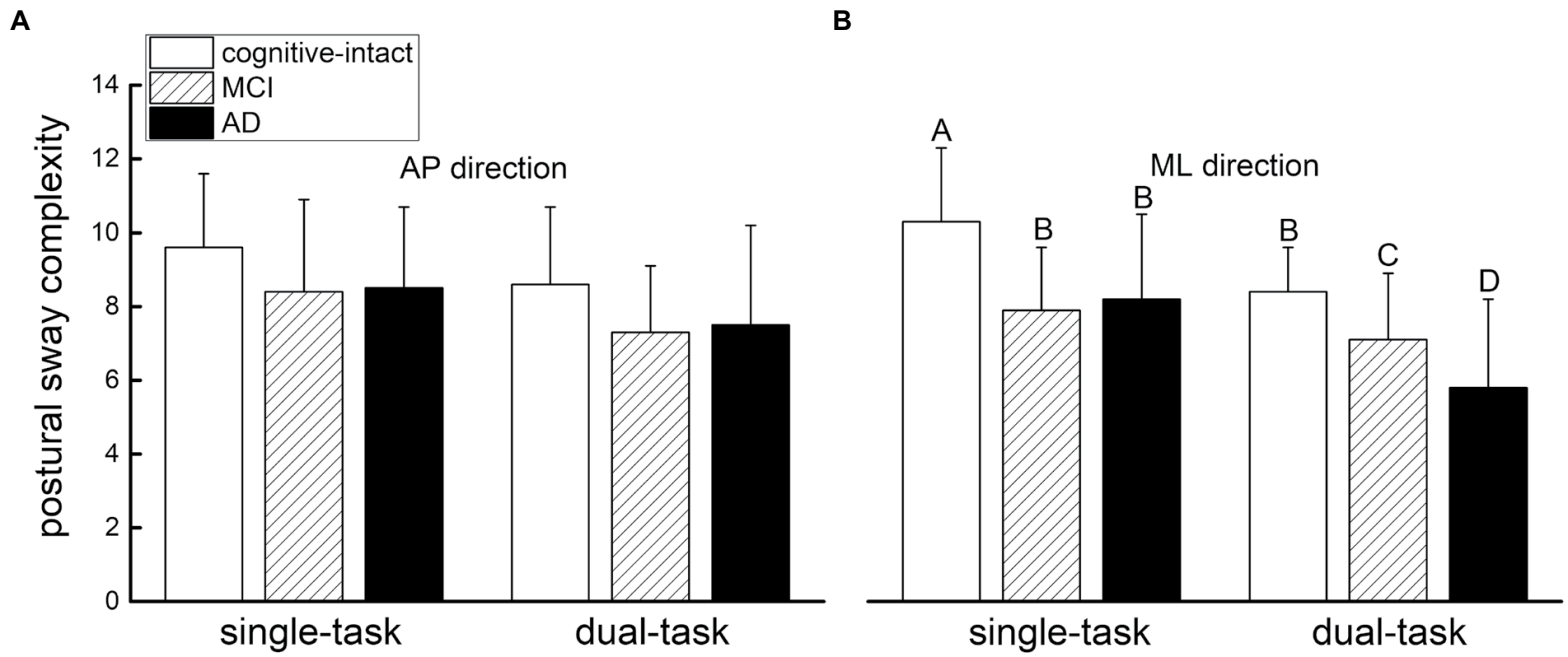
Postural sway complexity in the anterior–posterior (AP) **(A)** and medial-lateral (ML) **(B)** directions within each group under single-task (i.e., quiet standing) and visual-search dual-task condition. Repeated-measures ANOVA models revealed significant main effects of group (*F* > 4.8, *p* < 0.01) and condition (*F* = 7.7, *p* < 0.007) on both AP and ML sway complexity. A significant interaction was observed between group and condition for ML sway complexity (*F* = 3.7, *p* = 0.03), but not for AP sway complexity (*F* = 0.22, *p* = 0.88). Compared to single-task sway complexity within cognitively-intact group, greater reduction in complexity was observed with increased severity of cognitive impairment (i.e., MCI and AD) and during performance of the visual-search dual-task. Different letters in the figure indicate significant differences of factor means based upon *post-hoc* testing.

Repeated measures ANOVA revealed significant main effects of group (AP: *F* = 4.8, *p* = 0.01; ML: *F* = 11.4, *p* < 0.001) and condition (AP: *F* = 7.7, *p* = 0.007; ML: *F* = 7.7, *p* < 0.001) on both AP and ML sway complexity. *Post-hoc* analyses revealed that: (1) compared to the cognitively-intact group, the MCI and AD groups had a lower ML (Figure 1A) and AP (Figure 1B) postural sway complexity; and (2) across participants, both AP and ML complexity were significantly lower during performance of the visual-search dual-task condition compared to single-task condition. Uniquely, a significant interaction was observed between group and condition in ML sway complexity (*F* = 3.7, *p* = 0.03), but not in AP sway complexity (*F* = 0.22, *p* = 0.88). Specifically, compared to the single-task sway complexity within the cognitively-intact group, greater reduction in complexity was observed with the increased severity of cognitive impairment (i.e., MCI and dementia) and during performance of the visual-search dual-task; the complexity of dual-task condition within AD group was lowest (Figure 1). No other significant differences were observed.

**Figure 2:**
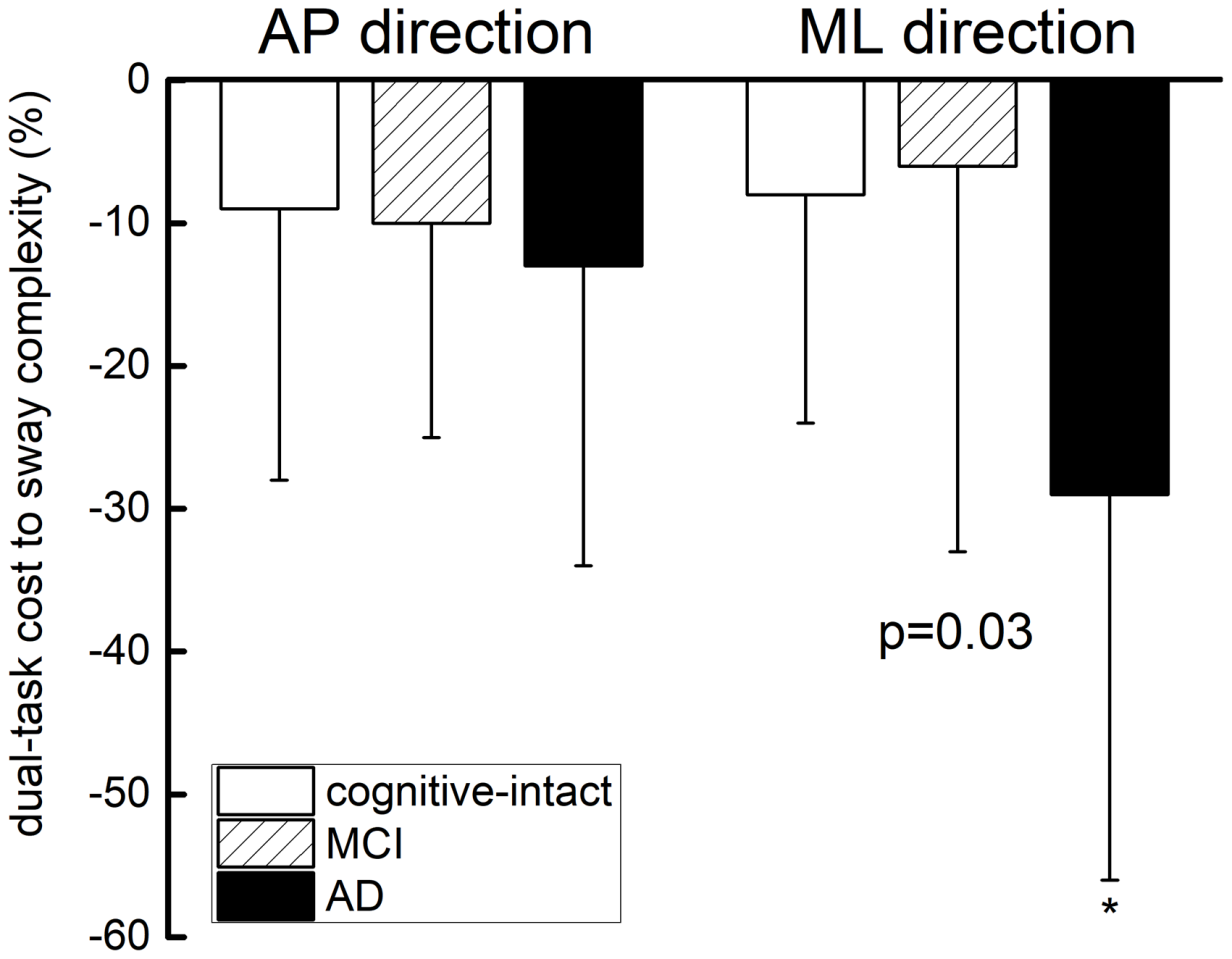
Effect of cognitive impairment on the dual-task cost to postural sway complexity in the anterior–posterior (AP) and medial-lateral (ML) directions. The dual-task cost to sway complexity was calculated as the percent change from the single-task (i.e., quiet standing) to visual-search dual-task condition for each direction separately. One-way ANOVA revealed a main effect of group (*F* = 3.67, *p* = 0.03) such that the AD group had greater dual-task cost to ML sway complexity as compared to cognitively-intact and MCI groups. This difference, however, was not observed in the dual-task cost to AP sway complexity.

One-way ANOVA revealed a main effect of group (*F* = 3.67, *p* = 0.03) such that the AD group had a greater percent decrease in the dual-task cost to ML sway complexity as compared to cognitively-intact and MCI groups, independent of age, education, and status of type 2 diabetes and hypertension (Figure 2). This difference, however, was not observed in the dual-task cost to AP sway complexity (*F* = 0.25, *p* = 0.78).

### The effect of cognitive impairment on traditional postural sway metrics

Repeated measures ANOVA models revealed a main effect of group for both sway path length (*F* = 9.8, *p* = 0.0003) and sway area (*F* = 7.1, *p* = 0.002). *Post-hoc* analyses revealed that compared to the cognitively-intact and MCI groups, those with AD had significantly greater sway path length and sway area irrespective of task condition. Neither the main effect of condition nor the interaction term for group and condition reached significance for any metric (*F* < 0.57, *p* > 0.57). The main effect of group did not reach significance for either the dual-task cost to sway path length (*F* = 1.77, *p* = 0.19) or sway area (*F* = 0.98, *p* = 0.39).

### The association between visual-search task performance and postural sway metrics

**Figure 3:**
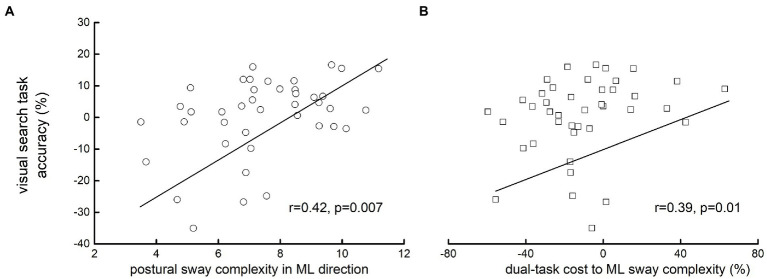
The association between visual-search performance and medial-lateral (ML) postural sway complexity **(A)** and the dual-task cost to this sway metric **(B)**. Visual-search task accuracy (%) was calculated as the number of target letters reported by the participant divided by the total number of target letters within the amount of letters scanned, multiplied by 100. Linear regression models showed that participants with lower ML sway complexity (*r* = 0.42, *p* = 0.007) and/or greater dual-task costs to ML sway complexity (*r* = 0.39, *p* = 0.01) would have significantly lower accuracy of visual-search task.

Accuracy on the visual-search task did not differ between groups (*F* = 1.49, *p* = 0.24). Across all participants, visual-search task accuracy was correlated with ML sway complexity during performance of the visual-search task (*r* = 0.42, *p* = 0.007) as well as the dual-task cost to ML sway complexity (i.e., greater percent reduction in sway complexity from single- to dual-task condition) (*r* = 0.39, *p* = 0.01; Figure 3). In each case, participants with lower dual-task ML sway complexity and/or greater dual-task cost to ML complexity had worse visual-search performance. This relationship was independent of age, group, education, type 2 diabetes, and hypertension. No significant associations were observed between visual-search accuracy and AP sway complexity (*r* = 0.1, *p* = 0.36) or dual-task cost to AP sway complexity (*r* = 0.02, *p* = 0.89). Moreover, no within-group associations between task accuracy and postural sway complexity outcomes were observed (*p* > 0.52).

Secondarily, visual-search performance was associated with the dual-task cost to sway path length, that is, those with greater increase in sway path length had worse visual-search accuracy (*r* = 0.3, *p* = 0.03). Visual-search performance was not significantly correlated with sway area or path length within either single- or dual-task condition, or the dual-task cost to sway area (*r* < 0.1, *p* > 0.42).

## Discussion

In general, compared to cognitively-intact older adults, those with MCI or AD exhibited lower postural sway complexity under both single- and dual-task conditions. The AD group demonstrated relatively greater dual-task cost to ML sway complexity as compared to both the cognitively-intact and MCI groups. Moreover, across all groups, lower ML postural sway complexity during the visual-search task condition (i.e., dual-task) was associated with lower task performance (i.e., accuracy in identifying the target letters). These results suggest that those with AD-related cognitive impairment exhibit a relative degradation in sway complexity and a greater loss of sway complexity when performing an attention-demanding cognitive task, as captured by MSE.

The function of physiological systems, including the postural control system, is regulated by multiple inputs and underlying components that interact with one another across multiple temporal scales (Costa et al., 2002; Lipsitz, 2002; Jiang et al., 2013). The quantity and quality of these components can be altered by both intrinsic (e.g., age, disease) and extrinsic factors (e.g., task specificity and demand). For example, Manor et al. (2010), investigated the effects of chronic sensory impairments on postural sway complexity during performance of a verbal serial subtraction dual-task paradigm (Manor et al., 2010). The authors determined that lower standing sway complexity during quiet standing (i.e., single-task) was linked to diminished vision acuity and foot sole somatosensation in older adults. Additionally, sway complexity decreased during dual-tasking, further highlighting the decrease in the ability of postural control system to adapt to perturbations/stressors (Manor et al., 2010). Such results are supported by separate studies that linked lower postural sway complexity to frailty (Lipsitz, 2002, 2004; Kang et al., 2009) and increased risk for future falls (Zhou et al., 2017). The current study provided novel evidence that older men with cognitive impairment (i.e., MCI or AD) had lower ML and AP standing postural sway complexity, compared to the cognitively-intact group. Moreover, the results revealed that older adults with poorer cognitive function (i.e., AD group) tended to have greater dual-task cost to postural sway complexity revealing a degradation of the physiological system’s feedback loop underpinning postural adaptation to attention-demanding visual stressors. Longitudinal studies are warranted to determine if the complexity of the postural control system during a visual-search paradigm can predict the probability of transitioning from cognitively-intact to MCI and/or MCI to AD, a pathological event that could trigger earlier interventions.

Both MCI and AD have been linked to a reduced capacity to maintain standing balance, especially when performing a concurrent task. In conjunction with the need to reallocate cognitive resources, the visual-search task used in the current protocol imposed external constraints on the standing postural control. While both sway complexity and traditional measures captured the effects of group (i.e., cognitive impairment), the effects of task condition (i.e., dual-tasking) on postural control was only captured by sway complexity. Participants across all three groups had lower AP and ML postural sway complexity during performance of the visual-search dual-task, compared to the single-task condition. This may suggest that although traditional sway measures can capture the changes in postural control on single scale, the changes in the dynamics of postural control over multiple scales as induced by subtle neurophysiological factors can only be captured by sway complexity. This is consistent with previous studies that demonstrated in those who were healthy at baseline, lower complexity of one given physiological system can capture the micro or subtle changes in the regulation of this system and is, thus, linked to increased risk of conditions in the near future (Zhou et al., 2017; Ma et al., 2021). For example, we observed in our previous study that older adults who had lower postural sway complexity at baseline, especially in the dual-task condition, would suffer significantly greater fall incidence in the next 48 months (Zhou et al., 2017). Taken together, these findings indicate that sway complexity can serve as a novel marker to characterize the subtle age- or disease-related changes in elements underlying postural control and assist in the health management of these elements to enable better functioning (Lipsitz, 2004; Busa and van Emmerik, 2016).

The percent change in the dual-task cost to ML sway complexity, and not AP sway complexity, was lower in those with AD, indicating that ML sway may be particularly sensitive to the interference on postural control from stressors (i.e., visual-search dual-tasking), and potentially to the incidence of risk events. Previous studies report that ML sway is closely associated with the risk of event (e.g., falls) in older adults and patient populations (Stel et al., 2003; Błaszczyk and Orawiec, 2011; Park et al., 2014; Sun et al., 2019) and the changes in ML complexity is also associated with the changes in functions pertaining to postural control in vulnerable populations (Etzelmueller et al., 2020). For example, in our previous study, we observed that postural sway complexity in ML direction was particularly sensitive to the changes in somatosensation of foot soles as induced by sub-threshold vibrations (Zhou et al., 2016). Importantly, studies have suggested that the loss of complexity is not an obligatory consequence, but instead, can be optimized with appropriate interventions that simultaneously augment the quantity and/or quality of the inputs to the postural control system and the functional components underlying the standing postural control (e.g., Tai Chi and resistance training) (Chen and Jiang, 2014; Wayne et al., 2014). Therefore, it may be particularly beneficial for rehabilitative strategies to target and assess the complexity in the ML direction.

This study is the first to explicitly explore postural sway complexity during performance of a visual-search task in older adults with and without cognitive impairments. With a small sample size, we were able to detect group and condition differences in postural sway complexity during the performance of visual-search protocol. Those with cognitive impairment displayed lower postural sway complexity and greater dual-task cost to postural sway complexity, compared to those that were cognitively-intact. Our results also revealed a link between postural sway complexity and task performance. The sample comprised of all men, because the participants were recruited from a Veteran community of which the number of men is much higher than women. Since this work is a secondary analysis of a parent study, we cannot perform the justification of participant sample size, and the number of participants in each group was different. Additionally, prior research has shown a link between loss of complexity and frailty (Lipsitz, 2002, 2004; Kang et al., 2009), but the current study did not assess for frailty, nor query for the information regarding the lived experiences of Veterans or balance confidence of participants, which may influence their sway performance. Therefore, future well-powered, sex-balanced studies with more explicit characterization of participants are needed to confirm our results for the general population. Our study consisted of a visual-search task with one level of difficulty. It would be worthwhile to explore the effects of varying levels of visual task difficulty (as there might have been a ceiling effect for some participants) as well as other types of task constraints on postural sway complexity. Nevertheless, this study demonstrates the effects of cognitive impairment and task constraints on standing postural sway complexity during a visual-search task. This complexity metric may in turn serve as a novel cognitive-motor biomarker to help predict and/or assess the loss of perception-action functionality in older adults.

## Data availability statement

The raw data supporting the conclusions of this article will be made available by the authors, without undue reservation.

## Ethics statement

The studies involving human participants were reviewed and approved by the Institutional Review Board of the Minneapolis VA Medical Center. The patients/participants provided their written informed consent to participate in this study.

## Author contributions

JZ and AJ designed the study. JM, MW, and AJ collected the data. JZ, BM, and AJ analyzed the data and performed the statistical analyses. JZ, BM, JM, MW, and AJ interpreted the results and prepared the manuscript. All authors contributed to the article and approved the submitted version.

## Funding

This work was supported by the National Institutes of Health (grant AG051766) awarded to AJ. BM and JZ are supported by the National Institutes of Health (grant number AG059089-01). JZ is supported by the grant from the National Institutes of Health (AG075180-01).

## Conflict of interest

The authors declare that the research was conducted in the absence of any commercial or financial relationships that could be construed as a potential conflict of interest.

## Publisher’s note

All claims expressed in this article are solely those of the authors and do not necessarily represent those of their affiliated organizations, or those of the publisher, the editors and the reviewers. Any product that may be evaluated in this article, or claim that may be made by its manufacturer, is not guaranteed or endorsed by the publisher.
